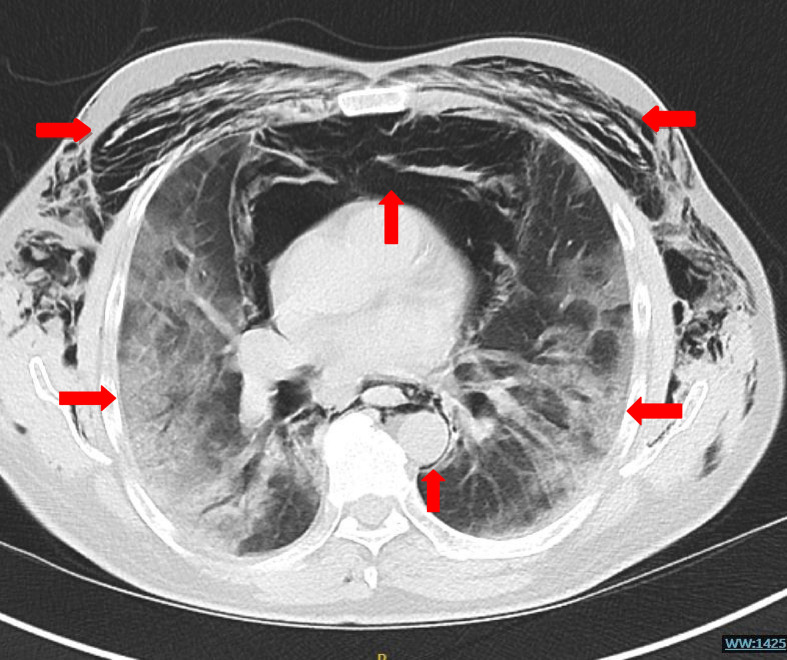# Pneumomediastinum in association with Covid-19: A less commonly considered differential diagnosis for worsening respiratory failure

**DOI:** 10.5339/qmj.2023.4

**Published:** 2023-01-02

**Authors:** Khalid Rashid, Muhammad Aamir Waheed, Yahya Khan, Adnan Afridi, Farrukh Ansar, Abdelnaser Elzouki

**Affiliations:** ^1^James Cook University Hospital, UK.; ^2^Hamad Medical Corporation, Qatar. E-mail: mawaheedhmc@yahoo.com ORCID: E-mail: 0000-0002-9056-5245; ^3^Mardan Medical Complex, Pakistan.; ^4^Northampton General Hospital NHS Trust, Acute Medicine.; ^5^King Abdullah Teaching Hospital, Pakistan.; ^6^Weill Cornell Medicine - Qatar.

**Keywords:** COVID-19, Respiratory system, Drugs and medicines, Pneumomediastinum

## Abstract

We have reported here two cases of coronavirus disease-2019 (COVID-19) patients aged 29 and 68 years who were diagnosed with pneumomediastinum (PM). PM is a rare complication that is being reported in association with COVID-19. Patients with COVID-19 can present with a variety of etiologies that make them vulnerable to PM. Respiratory complications due to COVID-19 are widely known, and it presents as mild to severe and critical illness. Spontaneous PM is a known complication of COVID-19. Despite seeming to be a lesser-known condition, PM can have a significant impact on disease progression and prognosis. We have presented here two contrasting cases of PM. The first patient was young and with moderate COVID-19 pneumonia and PM, while the second one was an old man with severe COVID-19 pneumonia manifestations. Both patients were diagnosed with PM, but their outcomes were completely different.

## Background

Across the world, millions of people were affected by the coronavirus disease-2019 (COVID-19) infection and thousands lost their lives to this illness.^1^ This infection is caused by an RNA virus known as “severe acute respiratory syndrome coronavirus-2 (SARS-CoV-2).”^2^ It primarily affects both the upper and lower respiratory tracts, resulting in a spectrum of symptoms ranging from sore throat and loss of taste and smell to cough, respiratory failure, and even multiorgan dysfunction.^3^ Most patients that develop severe symptoms are admitted to different hospital settings, including the intensive therapy unit. These patients are likely to develop complications related to COVID-19 and the resulting interventions. These interventions include both invasive and noninvasive ventilatory support. As per the literature, patients with the severe disease develop acute respiratory distress syndrome (ARDS).^4^ Moreover, the secondary bacterial infection has been attributed to causing severe respiratory distress and prolonging the symptoms. Hypercoagulability can lead to venous thromboembolism (VTE) and possibly pulmonary embolism (PE) can lead to respiratory failure.^5^ In addition to these more common causes of severe respiratory symptoms, a few uncommon conditions can give rise to similar symptoms, including tension pneumothorax and PM, which is the presence of air in the mediastinum. PM may occur spontaneously or because of (baro) trauma. Spontaneous pneumomediastinum (SPM) was previously associated with various infections, such as tuberculosis and influenza; nevertheless, as evident from the literature review, there is now an increasing number of cases reported secondary to COVID-19.^6-12^ PM is mostly self-resolving, but it can prove to be life-threatening if it results in tension pneumothorax.^13^ We have presented here contrasting clinical cases of a 29-year-old man and a 68-year-old man who developed spontaneous and secondary PM in association with COVID-19.

## CASE 1

We have presented here the case of a 29-year-old man who arrived at the emergency department with the chief complaints of worsening shortness of breath for the past 5 days. On initial assessment, he was a lean young man who could sit on his own. However, he was breathless and unable to speak in complete sentences. His general physical examination was remarkable for oxygen saturation of 84% on room air, heart rate of 110 beats per minute, and a raised respiratory rate of 30/minute. His recorded body temperature was in the normal range. His respiratory examination revealed bilateral crepitation, although the trachea was central and the percussion notes were dull. The rest of the systemic review was unremarkable.

The patient had no background of any comorbidities or chronic illnesses. He was an active young gentleman who was otherwise fit and well. His family history of interest included VTE in a first-degree relative. The patient stated that he was single, lived alone, and managed his daily routine by himself. He denied any history of cigarette smoking but drank alcohol occasionally. There was no positive history of illicit drug use. Furthermore, there was no recent travel history, and he was not vaccinated against COVID-19.

### Investigations

**Figure 1.**
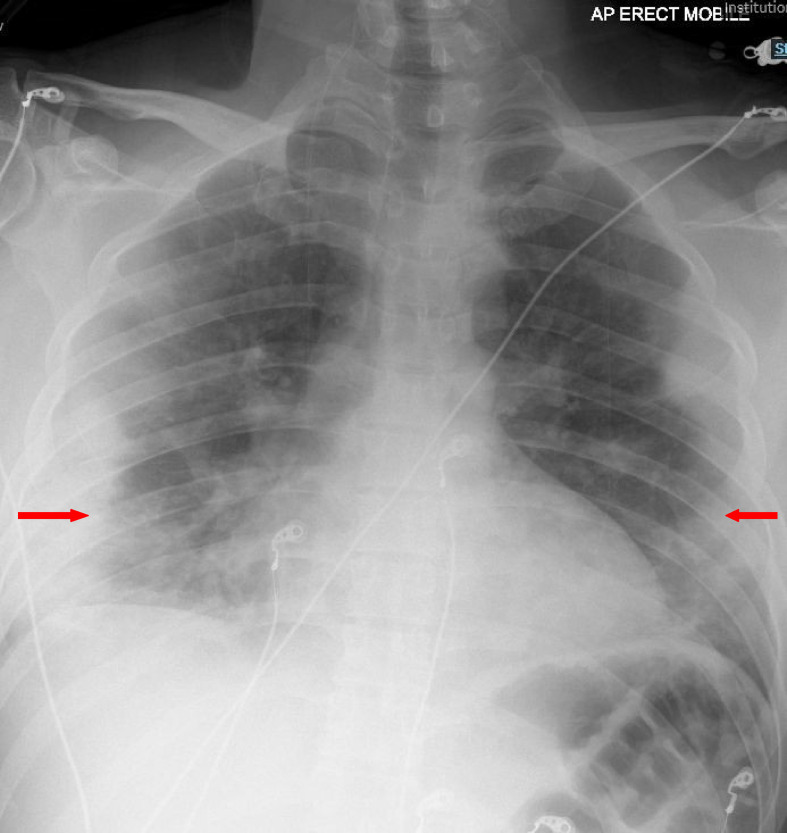


The patient tested positive for COVID-19 on the reverse transcriptase-polymerase chain reaction (RT-PCR). His initial chest X-ray (CXR) revealed coalescing patchy consolidations in the peripheral and mid-to-lower zones, which were consistent with the symptoms of mild COVID (Figure 1). His initial levels were as follows: D-dimers, 1392 ng/mL; lactate dehydrogenase (LDH), 865 U/L; C-reactive protein (CRP), 39 mg/dL. Other laboratory results were remarkable for a white cell count of 3.4, a neutrophil count of 2.68, and a lymphocyte count of 0.64. His urea and electrolytes panel were unremarkable, except for the low levels of sodium (126 mmol/L). The initial venous blood gas obtained on admission revealed a pH of 7.421, PO2 of 6.40 kPa, PCO2 of 5.03 kPa, and bicarbonate level of 24.3 mmol/L.

### Treatment and Outcome

**Figure 2.**
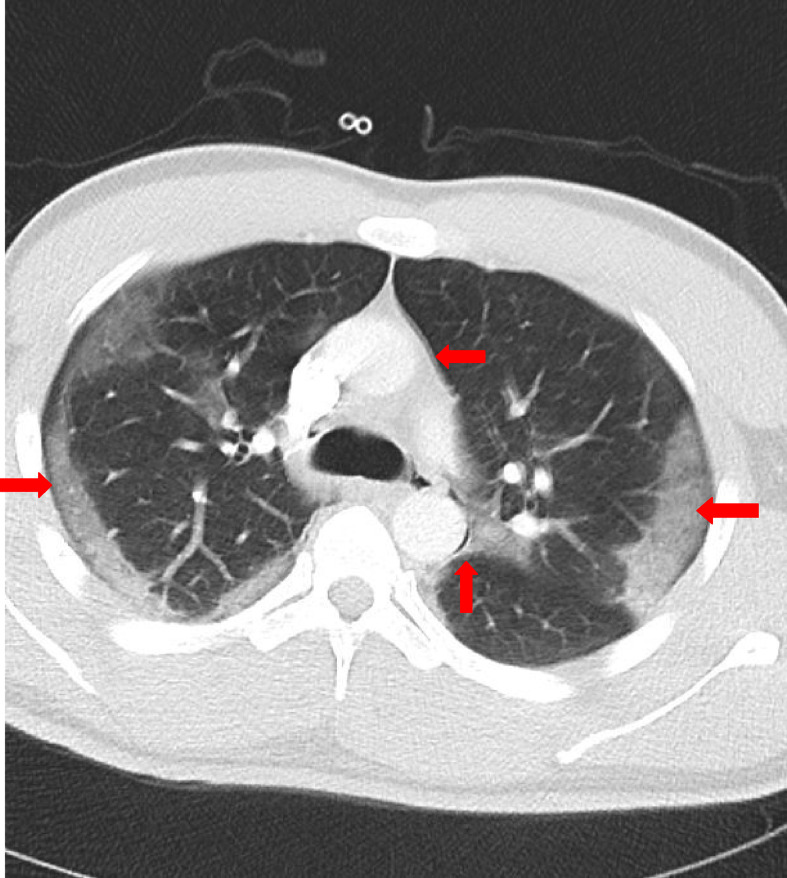


Owing to his condition, the patient was admitted to the acute medical unit and initiated on intravenous coamoxiclav, oral clarithromycin, and dexamethasone, in addition to 4 L of oxygen through a nasal cannula. During his stay, he was advised to be self-prone daily. With the aforementioned management plan in place, the patient was found to be settled and feeling better, which was ascertained by his improving respiratory and heart rates. However, his oxygen requirement increased gradually, and he became more tachycardiac and tachypneic on the second day of admission (DOA). At this point, reassessment by the ward team led to a repetition of the measurement of his D-dimers to rule out PE. Moreover, he was administered a high percentage of oxygen through a mask (40%). Subsequently, his D-dimer was found to be higher (4000 ng/dL) than that at the time of this admission (1392 ng/dL). In addition to the other clinical features, this observation raised suspicion about PE. Therefore, a treatment dose of low-molecular-weight heparin for PE was administered, and a computed topography-pulmonary angiogram (CTPA) was performed on the third DOA, albeit no embolus was identified in his pulmonary arteries. Nevertheless, the presence of air density in the mediastinum along with bilateral subcutaneous emphysema was noted in addition to peripheral ground-glass consolidation. These findings were consistent with PM on the background of COVID-19 pneumonitis. Chest CT was performed, which consolidated the diagnosis of PM along with COVID-19 and esophageal pathology as the cause for the presence of air in the mediastinum (Figure 2). On the sixth DOA, the patient started to show improvement. His oxygen saturation was maintained at 98–99% on 28% oxygen, and his other vitals were in the normal range. On the seventh DOA, his supplemental oxygen requirement improved, and his saturation was maintained at 97% on 2–3 L of oxygen. We, accordingly, decided to keep the patient under observation for another 24 h. On the eighth DOA, he was found to be feeling well and not requiring further oxygen supplementation, and he was discharged on oral antibiotics.

## CASE 2

We have presented here the case of a male patient aged 68 years who presented with shortness of breath, dry cough, and mild chest discomfort on the ninth day after his positive COVID-19 PCR report. He was in respiratory distress on initial examination, as he was tachypneic (respiratory rate of 30) and could not speak in complete sentences. Moreover, the oxygen saturation in the room air was 85%, while his heart rate was 82 bpm, blood pressure was 114/63, and body temperature was 37°C. His respiratory examination revealed bilateral crackles on both lung fields. He had a background of hypertension and hypercholesterolemia. He was an active middle-aged man with a clinical frailty score of 3.^14^ He was a nonsmoker, had never taken any form of alcohol, and had not received vaccination against SARS-CoV-2.

### Investigations

**Figure 3.**
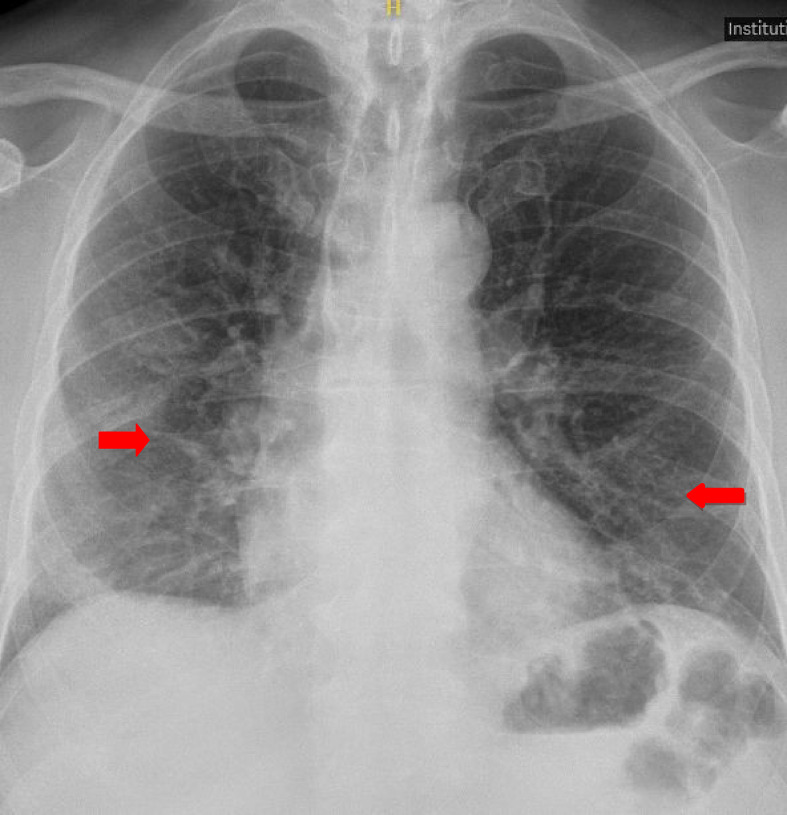


The patient was found positive for COVID-19 on RT-PCR. His initial CXR revealed bilateral patchy airspace consolidation in the peripheral mid and lower zones, which was consistent with the findings of COVID-19 pneumonia (Figure 3). His initial levels of D-dimers and CRP were 266 ng/mL and 43 mg/dL, respectively. The other laboratory studies revealed a white cell count of 3.4, a neutrophil count of 2.73, and a lymphocyte count of 0.35. The urea and electrolytes panel were found to be in the normal range.

### Treatment and Outcome

We initiated the patient on oral dexamethasone, intravenous coamoxiclav, and oral clarithromycin, as per the existing protocol. We also started him on 4 L of oxygen through a nasal cannula. The patient’s oxygen demand was increased to 15 L on the third DOA. However, on the fourth DOA, he developed hypoxia on 15 L of oxygen.

**Figure 4.**
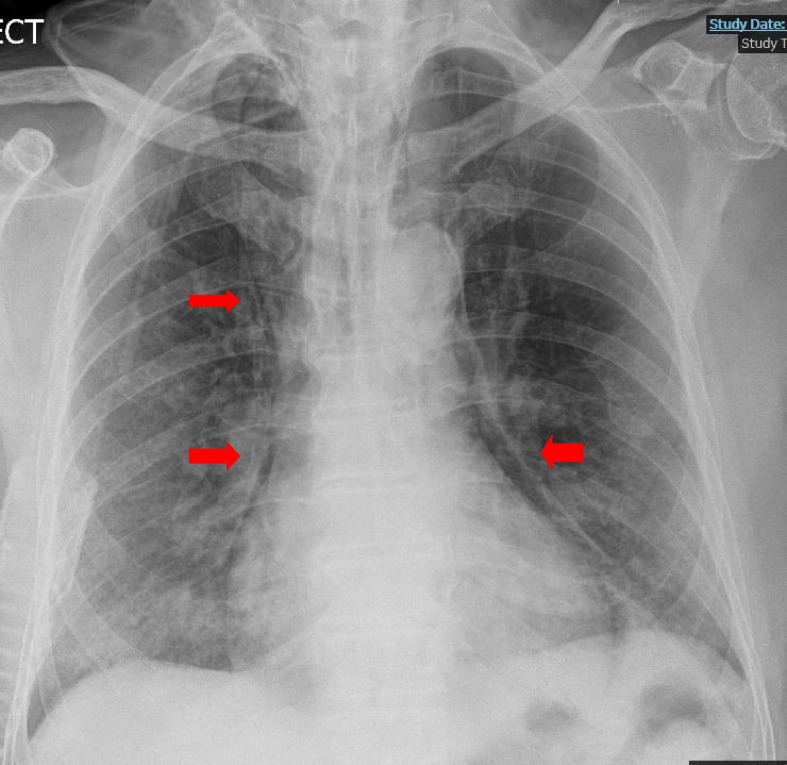


Therefore, the patient was put on continuous positive airway pressure (CPAP), which slowed his breathing rate and brought his oxygen saturation to 94%. On the seventh DOA, he maintained his saturation at the lowest possible settings of the CPAP machine. Accordingly, it was decided to wean him off CPAP to 15 L of oxygen through a nonrebreather mask. At this time, the patient complained of chest pain, for which he was subjected to an ECG, a complete set of blood tests, and a CXR. His blood tests revealed a rising D-dimmer level (5221 ng/mL). The cardiac cause of his chest pain was excluded based on the normal troponin levels and normal sinus rhythm on ECG. The radiologists reported his CXR as “air lucency along the lateral wall of the upper trachea, which represents a subtle PM.” His chest pain worsened relative to his presenting symptoms, which were now associated with facial and periorbital swelling. His examination revealed neck and chest wall crepitus. Repeat CXR affirmed the diagnosis of subcutaneous emphysema in the neck and bilateral chest walls in addition to PM and patchy consolidation due to COVID-19, which were all found to be advancing (Figure 4). These events coincided with his worsening hypoxia and severe dysphagia secondary to PM and progressive ARDS. Urgent thorax CT exhibited extensive surgical emphysema throughout the soft tissues of the thorax and lower neck. It also showed peripheral ground-glass changes throughout both lungs with patchy consolidations. The extensive PM was worse than that on the previous CXR (Figure 5), accordingly, CTPA was ruled out. As a result, the lung multidisciplinary team care decided to amend his escalation plan to ward-based care. The team believed that CPAP, NIV, and mechanical ventilation were not appropriate interventions to alleviate his ailments. It was agreed to manage his PM and subcutaneous emphysema conservatively. His oxygen saturation was persistently below the target, and his consciousness level was reduced. Eventually, after spending 22 days in the hospital, he passed away peacefully.

## Discussion

We have presented here two contrasting cases of PM. The first patient was young with moderate COVID-19 pneumonia and PM, while the second case was a severe COVID-19 pneumonia manifestation. Both patients were diagnosed with PM, albeit the outcome was completely different. In the first case, the patient did not have any precipitating or predisposing factors in his medical history, such as illicit drug use or asthma. As the patient became more tachypneic and tachycardic on the second DOA, PE was considered the leading cause, for which testing of D-dimers and CTPA were advised. The CT PA was performed on the third DOA, which ruled out PE and indicated the presence of SPM. This case suggested a diagnostic delay; a simple bedside CXR, if done on time, would have helped diagnose SPM. As Spiezia et al. reported, COVID-19 is associated with hypercoagulable states, and thus PE is a liable diagnosis in cases of acute degeneration.^15^However, with increasing numbers of reported cases of SPM in COVID-19, SPM should be added to the list of possible differential diagnoses. As our case report indicates, clinicians should have a low threshold for diagnostic and readily available radiological imaging such as CXR and chest CT for timely diagnosis of SPM.

The second case patient was an elderly gentleman whose condition deteriorated gradually, and his oxygen requirements also increased gradually until he was started on CPAP on the fifth DOA. Subsequently, he developed symptoms of chest pain, dysphagia, and facial swelling. These symptoms indicated the development of subcutaneous emphysema along with PM, which was confirmed later by CXR and CT chest. Eventually, CPAP was discontinued, and palliative management was initiated. As this case demonstrates, complications arising from PM and other associated conditions may ultimately prove fatal. Thus, prompt diagnosis and management of PM in COVID-19 patients are critical.

Although the exact mechanism for the occurrence of PM is yet to be determined, the Macklin effect could explain its pathophysiology.^16,17^ It postulates that PM occurs because of the development of a pressure gradient between an alveolus and the surrounding lung interstitial tissues, which causes a ruptured alveolus. Subsequently, the air leaks into the mediastinum. Damage to the alveolar tissues can be secondary to inflammatory changes to its wall or factors that precipitate high intra-alveolar pressure, such as high-flow oxygen through mechanical and noninvasive ventilation. It may commonly present with retrosternal chest pain, neck pain, swelling, facial swelling, dyspnea, dysphagia, and weakness. Diagnosis is usually made based on a CXR, while a chest CT scan is a gold standard for assessing progression.^18^ PM is generally considered a benign condition, and its treatment is usually conservative, with a focus on treating the underlying condition and avoiding any maneuvers that may increase intrathoracic pressure.^19^

According to the literature, an increasing number of cases of PM has been reported secondary to COVID-19.^6-12^ The exact mechanism and pathophysiology of PM in these patients are unknown. However, COVID-19-associated pneumonia leading to ARDS can cause alveolar damage and a subsequent rupture, thereby precipitating the likelihood of SPM as explained earlier. Dry cough spells, a common feature of COVID-19 disease, can lead to increased intrathoracic pressure, causing PM.

Finally, secondary PM can hypothetically manifest in patients on artificial ventilation as a result of barotrauma. This phenomenon has only been reported a few times in patients with COVID-19. Thein et al. reported two cases of secondary PM in two elderly COVID-19 patients who underwent prolonged NIV therapy.^20^ Our second case highlights the importance of recognizing this rare complication considering that a significant number of COVID-19 patients end up receiving high-pressure ventilation through CPAP as per the British Thoracic Society Guidelines, which puts them at risk for clinical deterioration as a result of PM. Therefore, prompt knowledge about the signs and radiological investigation is critical, as PM can prove fatal.

### Learning Points

1. PM may occur in young as well as old patients.
2. PM should be considered as a differential diagnosis of worsening respiratory symptoms in COVID-19 patients in addition to more common causes.
3. CXR should be used as a first-line radiological investigation in COVID-19 patients with worsening symptoms as it aids in the diagnosis of multiple conditions, including PM. Clinicians should have a low threshold for performing radiological imaging such as chest CT in COVID-19 patients with a sudden increase in oxygen requirement.
4. NIV in COVID-19 patients can lead to SPM.

## Conclusion

PM is a rare complication of COVID-19 pneumonia with significant lung involvement, which may or may not require invasive ventilatory support. However, the coexistence of PM with a significant parenchymal lesion on CT suggests serious alveolar membrane degradation and, consequently, a probable deterioration of the clinical outcomes. On the other hand, when the lung lesions are not severe, the clinical course appears to be better with an improved prognosis. Future research should include more numbers of patients to establish a correlation between PM and a potential prognostic factor.

## Figures and Tables

**Figure 5.**